# Extracellular overhydration linked with endothelial dysfunction in the context of inflammation in haemodialysis dependent chronic kidney disease

**DOI:** 10.1371/journal.pone.0183281

**Published:** 2017-08-22

**Authors:** Nicos Mitsides, Tom Cornelis, Natascha J. H. Broers, Nanda M. P. Diederen, Paul Brenchley, Frank M. van der Sande, Casper G. Schalkwijk, Jeroen P. Kooman, Sandip Mitra

**Affiliations:** 1 Division of Cardiovascular Sciences, School of Medical Sciences, Faculty of Biology, Medicine and Health, The University of Manchester, Manchester Academic Health Science Centre, Manchester, United Kingdom; 2 Nephrology Department, Central Manchester University Hospital NHS Foundation Trust, Manchester Academic Health Science Centre, Manchester, United Kingdom; 3 NIHR Devices for Dignity Healthcare Technology Co-operative, Royal Hallamshire Hospital, Sheffield, United Kingdom; 4 Jessa Hospital, Hasselt, Belgium; 5 Department of Internal Medicine, Division of Nephrology, Maastricht University Medical Center, Maastricht, Netherlands; 6 NUTRIM School of Nutrition and Translational Research in Metabolism, Maastricht University, Maastricht, Netherlands; 7 CARIM School for Cardiovascular Diseases, Maastricht University, Maastricht, Netherlands; The University of Tokyo, JAPAN

## Abstract

**Background:**

Haemodialysis (HD) patients are predisposed to dysregulated fluid balance leading to extracellular water (ECW) expansion. Fluid overload has been closely linked with outcome in these patients. This has mainly been attributed to cardiac volume overload, but the relation between abnormalities in fluid status with micro- and macrovascular dysfunction has not been studied in detail. We studied the interaction of macro- and microvascular factors in states of normal and over- hydration in HD-dependent CKD.

**Methods:**

Fluid compartments [total body water (TBW) and ECW] and overhydration index (OH) were measured with Multifrequency bio-impedance (BCM). Overhydration was defined as OH/ECW>7%. Overhydration was also assessed using the ECW/TBW ratio. Macrocirculation was assessed by pulse-wave velocity (PWV) and mean arterial pressure (MAP) measurements while microcirculation through sublingual capillaroscopy assessment of the Perfused Boundary Region of the endothelial glycocalyx (PBR 5-25mcg). A panel of pro-inflammatory and vascular serum biomarkers and growth factors was analysed.

**Results:**

Of 72 HD participants, 30 were in normohydration (N) range and 42 overhydrated according to the OH/ECW ratio. Average ECW/TBW was 0.48±0.03. Overhydrated patients had higher MAP (122.9±22.5 v 111.7±22.2mmHg, p = 0.04) and comorbidities (median Davies score 1.5 v 1.0, p = 0.03). PWV (p = 0.25) and PBR 5-25mcg (p = 0.97) did not differ between the 2 groups. However, Vascular Adhesion Molecule (VCAM)-1, Interleukin-6 and Thrombomodulin, and reduced Leptin were observed in the overhydrated group. Elevation in VCAM-1 levels (OR 1.03; 95% CI 1.01–1.06; p = 0.02) showed a strong independent association with OH/ECW>7% in an adjusted logistic regression analysis and exhibited a strong linear relationship with ECW/TBW (Bata = 0.210, p = 0.03) in an also adjusted model.

**Conclusion:**

Extracellular fluid overload is significantly linked to microinflammation and markers of endothelial dysfunction. The study provides novel insight in the cardiovascular risk profile associated with overhydration in uraemia.

## Introduction

Despite advances in technologies and practice, outcomes in haemodialysis (HD) remain poor [1]. The role of dysregulated fluid balance in cardiovascular mortality and morbidity in HD-dependent chronic kidney disease (CKD) is well defined. Overhydration, in the context of uraemia, has been linked with hypertension, arterial stiffness [2] and left ventricular dysfunction [3] and is strongly associated with poor cardiovascular outcomes [3–5]. A large majority of HD patients also have coexistent diabetes (DM) and cardiovascular (CV) disease whilst uraemia in itself is widely considered a state of advanced atherosclerosis [6,7]. In HD-dependent patients, maintenance of optimal hydration parameters in the presence of multiple overlapping comorbidities, presents a significant clinical challenge.

There are distinct differences in the pathophysiology of macro- and microvascular disease [8]. The capillary endothelial glycocalyx, for example, is not only a marker of vascular injury [9] but may be an important component of salt and water homeostasis [10,11]. Microinflammation and endothelial dysfunction have been associated with both salt [12] and water overload [13]. This part of the circulation constitutes an important component of the CV system and could provide a missing link in the interplay between overhydration and systemic inflammation and their associated adverse outcomes in uraemia [13]. Addressing the knowledge gaps in our understanding of the relationship between overhydration, microcirculation and inflammation in this disease state would provide a vital insight in the management of hydration and CV risk in HD patients.

We investigated the clinical and biomarker characteristics of the circulation and its disposition in relation to hydration in uremic patients treated by HD.

## Materials and methods

We performed analyses in 72 HD participants recruited in Uremic Toxins, Cardiovascular Effects and Physical Activity in Intensive Haemodialysis (INTHEMO), a prospective multicentre international study. The study population was split evenly between participants receiving a 4hr, 3 times per week conventional HD (CHD) and those receiving extended HD (>12hr HD per week) regimes as part of their normal clinical care. The participating units included Maastricht University Medical Center and Heerlen Dialysis Unit in the Netherlands, Central Manchester University Hospitals NHS Foundation Trust in the UK and the Hasselt Dialysis Unit in Belgium. The study was approved by the Greater Manchester Research Ethics Committee (14/NW/1158) and the Medical Ethics Committee of Maastricht University Medical Center (NL35039.068.10). All consenting adult patients receiving their HD care at the participating centres were eligible for enrolment to the study. Informed written consent was obtained prior to participation to the study.

### Data collection

The past medical history and medication lists were collected directly from participants and through their electronic medical records. These were utilised to derive the Davies’ Comorbidity Score (DCS) [14]. HD prescription data were also collected to determine frequency and duration of HD treatment, the prescribed target weight and dialysis adequacy, expressed as standard Kt/V. All study measurements were performed at a single visit by 3 trained operators (1 for Manchester and 2 for the Netherlands and Belgium).

### Fluid status and compartmental distribution assessment

Total Body Water (TBW), ECW, Intracellular Water (ICW), Lean Tissue Index (LTI) and Fat Tissue Index (FTI), overhydration index (OH) were measured using multifrequency bioimpedance (Body Composition Monitor (BCM^®^), Fresenius Medical Care, Germany). Measurements were performed before the mid-week HD session for participants receiving in-centre HD and for participants performing home HD during the interdialytic interval. Overhydration was expressed as absolute OH and relative overhydration (OH/ECW) and participants were stratified according to this into normal hydration (OH/ECW < 7%) and overhydration (OH/ECW > 7%) [5,15,16]. In addition to OH/ECW, ECW/TBW ratio [17] was also used to assess volume expansion.

### Cardiovascular measurements

Macrocirculation was assessed by means of Pulse Wave Velocity (PWV) and Blood Pressure (BP) measured during the research visit and using 24hr Ambulatory Blood Pressure Monitoring (ABPM). Microcirculation was assessed by means of dark-field sublingual capillaroscopy (DFSC). PWV, BP and DFSC were measured at the same time as the hydration measurements.

#### Pulse wave velocity

PWV was measured during the research visit through carotid/femoral applanation tonometry using the SphygmoCor system (AtCor Medical, Australia) [18].

#### 24hr ambulatory blood pressure monitoring

24hr ABPM measurements were obtained using an oscillometric technique and the Mobil-O-Graph NG device (I.E.M GmbH, Germany). Measurements of BP were obtained at 30-minute intervals during the day and at 60-minute intervals during the night. Readings were analysed using the hypertension management software of the Mobil-O-Graph NG device. Incomplete measurements (not completing the 24hr period) were included for analysis provided data spreaded across the day.

#### Capillaroscopy

DFSC was used to measure microvascular endothelial injury. This was measured by means of Perfused Boundary Region (PBR) thickness of the endothelial glycocalyx and red blood cell (RBC) width using Sidestream Dark Field Imaging (GlycoCheck, Netherlands). The software records an adequate sample of high quality video frames of microvasculature, identifies all available microvessels and defines small vascular segments every 10 μm along their length. For each vascular segment 840 radial intensity profiles are obtained, which are tested for the presence of RBCs and signal quality, and then the RBC column widths (RBCW) are determined from these intensity profiles. The median RBCW and the outer edge of the RBC perfused lumen (Dperf) are determined from these measurements. The distance of the median RBCW value to the position of the outer edge of the RBC perfused lumen is defined as the PBR [PBR = (Dperf–RBCW)/2]. This methodology has been validated in both animal and human models [9,19–22] and the software has been used in a number of studies [23–26]. Both PBR and RBCW measurements has been associated with microvascular injury in CKD [19,27,28].

### Blood sampling and residual renal function assessment

All participants at the time of the research visit had their serum electrolytes, parathyroid hormone (PTH), β2-macroglobulin and serum biomarkers of the different pathophysiological pathways involved in the progression of vascular disease measures. These including markers of low-grade inflammation and endothelial dysfunction, angiogenic growth factors and hormones (Table 1). Analysis was performed using electro-chemiluminescence technology in a single and multi-array detection system (MesoScale Discovery SECTOR Imager 2400, Gaithersburg, Maryland, USA). All the measurements were performed in duplicate. Interassay and intra-assay variations for all the markers were <9%, with the exception of the interassay variation for bFGF (10.6%) and the interassay variation for IL-6 (16%).

**Table 1:** Vascular biology and pro-inflammatory biomarkers tested in the study.

Biomarker		MolecularWeight
**Vascular Endothelial Panel**	**Intracellular Adhesion Molecule (ICAM)-1 (ng/ml)**	75–115 kDa
	**Vascular Cell Adhesion Molecule-1 (VCAM-1) (ng/ml)**	100–110 kDa
	**E-selectin (ng/ml)**	107–115 kDa
	**P-selectin (ng/ml)**	86 kDa
	**ICAM-3 (ng/ml)**	95 kDa
	**Thrombomodulin (ng/ml)**	74 kDa
	**Matrix Metalloproteinase (MMP)-1 (ng/ml)**	52 kDa
	**MMP-3 (ng/ml)**	54 kDa
	**MMP-9 (ng-ml)**	82-92kDa
**Pro-inflammatory Panel**	**C- Reactive Protein (CRP) (μg/ml)**	110–144 kDa
	**Soluble Amyloid A (SAA) (μg/ml)**	12 kDa
	**Interleukin (IL)-6 (pg/ml)**	21–28 kDa
	**IL-8 (pg/ml)**	11 kDa
	**Tumour Necrosis Factor-alpha (TNF-α) (pg/ml)**	17 kDa
**Growth Factors**	**Basic Fibroblast Growth Factor (bFGF) (pg/ml)**	22-24kDa
	**Placenta Growth Factor (PIGF) (pg/ml)**	23 kDa
	**Soluble fms-like tyrosine kinase-1 (Flt-1) (pg/ml)**	100 kDa
	**Vascular Endothelial Growth Factor (VEGF) (pg/ml)**	38 kDa
	**Leptin (pg/ml)**	16–19 kDa
	**Insulin (pg/ml)**	6 kDa

kDa = kilo-Dalton, ml = millilitre, ng = nanogram, pg = picogram, μg = microgram.

Residual renal function was assessed by means of an interdialytic urine collection. Anuria was defined as urine volume < 100ml/day.

### Statistical analysis

Cohort characteristics and measurements were explored using descriptive epidemiology. Categorical variables were reported as percentages and ratios. Continuous variables were reported as mean (standard deviation) where distribution was normal and as median (minimum and maximum) when distribution was skewed. Normality of distribution was assessed using the Shapiro-Wilk method. Comparison of variables between hydration groups was performed using Pearson’s Chi^2^ test for categorical variables, t-test for continuous variables with normal distribution and Mann-Whitney U test for continuous variables with skewed distribution.

In order to investigate the relation between CV measurements, vascular and inflammatory biomarkers and overhydration, we built a model with OH/ECW as dependent variable. Possible predictors of OH/ECW as a dichotomous variable were initially assessed by means of univariate binary logistic regression. Variables with p<0.05 were entered in a forward sequential additive multivariate model. The model was adjusted for age, comorbidity and dialysis intensity defined as hours of HD per week. Models that included Leptin were also adjusted for FTI and LTI. Model best fit was assessed using ROC analysis. Possible predictors of ECW volume (ECW/TBW) expansion where initially assessed through univariate linear regression analysis. Predictor variables significant at the level of p<0.05 where included in a sequential additive multivariate linear regression model. Statistical analyses were performed by IBM SPSS Statistics, version 23 (IBM Corp., USA).

## Results

Seventy-two participants’ data were analysed and their demographic, CV and hydration profiles are summarised in Table 2. Of the study population, 36 participants were receiving CHD (<12hrs per week) while 36 were receiving extended regimes (EHD) (>12hrs per week) (demographic and measurement profiles of the 2 subgroups stratified by their OH/ECW presented in S1–S4 Tables). The mean OH in all subjects was 1.4±1.71L representing an OH/ECW of 7.06±8.76%. When OH/ECW was used to stratify the cohort population into overhydrated and not overhydrated, the categories were differentiated by ECW volume measurements (Table 3). Overhydrated patients had a higher BP and DCS (Table 3) but PWV and DFSC measurements did not differ between the 2 groups. Overhydration was characterised by markedly higher serum levels of Vascular Cell Adhesion Molecule (VCAM)-1, Thrombomodulin (TM) and Interleukin (IL) 6, and lower levels of Leptin (Table 3).

**Table 2:** Cohort demographic and dialysis profiles stratified by overhydration status.

Cohort Characteristics		Entire Cohort n = 72	No overhydration (OH/ECW < 7%)n = 30	Overhydration (OH/ECW > 7%)n = 42	Sig
**Age (year)**		57.3 (SD 14.3)	53.4 (SD 16.3)	60.1 (SD 12.2)	0.050
**Sex: Male**		55 (76.4%)	21 (70%)	34 (81%)	0.281
**Ethnicity**	**White**	57 (79.2%)	24 (80%)	33 (78.6%)	0.773
	**Black**	6 (8.3%)	2 (6.7%)	4 (9.5%)	
	**Asian**	8 (11.1%)	4 (13.3%)	4 (9.5%)	
	**Other**	1 (1.4%)	-	1 (2.4%)	
**Dialysis Vintage (months)**		74.3 (6–432)	39.5 (6–276)	46.5 (6–432)	0.304
**Residual Urine Output**		23 (31.9%)	8 (26.7%)	15 (35.7%)	0.417
**Previous Transplant**		27 (37.5%)	12(40%)	15 (35.7%)	0.711
**Diabetes Mellitus**		19 (26.4%)	7(23.3%)	12(28.6%)	0.619
**CVD**		20 (27.8%)	6 (20%)	14 (33.3%)	0.213
**Smoking**		10 (13.9%)	4 (13.3%)	6 (14.3%)	0.908
**Davies Comorbidity Score**		**1.0 (0–4)**	**1.0 (0–3)**	**1.5 (0–4)**	0.031*
**Number of BP medication**		1.0 (0–5)	1.0 (0–5)	1.5 (0–5)	0.091
**HD Parameters**	**Hrs per wk**	12.8 (10.5–42.5)	13.5 (12.0–42.5)	12.0 (10.5–32.0)	0.497
	**HD frequency per wk**	3 (3–6)	3 (3–5)	3 (3–6)	0.782
	**HD session length**	4 (2.5–9.0)	4 (4.0–8.5)	4 (2.5–9.0)	0.493
	**Standard Kt/v(n = 67)**	2.25 (1.69–3.69)	2.27 (1.87–3.66)	2.22 (1.69–3.23)	0.402
**PTH (pmol/L)**		26.6 (0.6–116.5)	23.1 (0.6–116.5)	27.3 (0.6–84.7)	0.879
**B2-microglodulin (mg/L)**		26.1 (SD 8.72)	26.9 (SD 8.39)	25.6 (SD 9.02)	0.542

BP = Blood Pressure, CVD = Cardiovascular Disease, ECW = Extracellular Water, HD = Haemodialysis, hr = hour, Kg = Kilogram, L = litre, mg = milligram, OH = Overhdration Index, pmol = picomole, PTH = Parathyroid Hormone, SD = Standard Deviation, Sig = Statistical Significance (p-value).

* Highlights Result with statistical significance at the level of p<0.05.

**Table 3:** Hydration and cardiovascular profiles stratified by the participants’ overhydration status.

Fluid and Cardiovascular Parameters		Entire Cohort n = 72	No overhydration (OH/ECW < 7%)n = 30	Overhydration (OH/ECW > 7%)n = 42	Sig
**Body Composition**	**OH (L)**	**1.5 (-1.8–6.7)**	**-0.1 (-1.8–1.3)**	**2.2 (0.9–6.7)**	<0.001*
	**OH/ECW (%)**	**8.08 (-12.8–27.8)**	**-0.8 (-12.8–6.7)**	**11.2 (7.0–27.8)**	<0.001*
	**TBW (L)**	37.59 (SD 7.41)	37.1 (SD 8.7)	37.9 (SD 6.5)	0.654
	**ECW (L/Kg weight)**	**0.23 (SD 0.03)**	**0.21 (SD 0.02)**	**0.24 (SD 0.03)**	<0.001*
	**ECW/TBW**	**0.48 (SD 0.03)**	**0.46 (SD 0.03)**	**0.50 (SD 0.03)**	<0.001*
	**Weight (Kg)**	81.0 (SD 15.9)	82.0 (SD 17.4)	80.3 (SD 15.0)	0.663
	**BMI (Kg/m2)**	27.21 (SD 4.9)	27.6 (SD 5.7)	27.1 (SD 4.2)	0.644
	**LTI (Kg/m2)**	12.6 (SD 2.6)	12.8 (SD 2.8)	12.5 (SD 2.4)	0.697
	**FTI (Kg/m2)**	13.6 (3.1–35.0)	14.3 (4.4–35.0)	13.5 (3.1–27.2)	0.337
**Visit BP(mmHg)**	**Systolic**	**139.6 (SD 28.9)**	**135.5 (SD 27.2)**	**146.1 (SD 28.7)**	0.023*
	**Diastolic**	75.6 (SD 14.5)	74.2 (SD 15.9)	76.6 (SD 13.5)	0.504
	**MAP**	**118.3 (SD 22.9)**	**111.7 (SD 22.2)**	**122.9 (SD 22.5)**	0.040*
**PWV (m/s) (n = 27:41)**		9.16 (SD 2.83)	8.7 (SD 2.33)	9.5 (SD 3.10)	0.252
**24hr BP(mmHg)(n = 27:37)**	**Average SBP**	132.3 (SD 23.9)	125.4 (SD 23.2)	137.2 (SD 23.6)	0.051
	**Average DBP**	80.7 (SD 15.4)	79.2 (SD 18.1)	81.8 (SD 13.2)	0.515
	**Average MAP**	97.9 (SD 17.1)	94.6 (SD 18.4)	100.3 (SD 15.9)	0.195
**Capillaroscopy**	**PBR 5–25**	1.97 (SD 0.27)	1.96 (SD 0.25)	1.97 (SD 0.28)	0.972
	**PBR 5–9**	0.97 (0.78–1.37)	0.97 (0.78–1.37)	0.95 (0.79–1.20)	0.958
	**PBR 10–19**	2.11 (SD 0.26)	2.14 (SD 0.24)	2.09 (SD 0.27)	0.419
	**PBR 20–25**	2.54 (SD 0.52)	2.46 (SD 0.42)	2.60 (SD 0.58)	0.256
	**Median P-50**	11.12 (SD 1.89)	11.42 (SD 2.04)	10.91 (SD 1.76)	0.272
**VascularBiology**	**ICAM-1 (ng/ml)**	428 (259–1345)	412.5 (259–612)	437.5 (269–1345)	0.465
	**VCAM-1 (ng/ml)**	**1066.4 (SD 302.5)**	**930.2 (SD 190.0)**	**1163.7 (SD 330.4)**	0.001*
	**E-selectin(ng/ml)**	10.9 (3.5–29.5)	11.2 (5.2–29.5)	10.5 (3.5–22.9)	0.219
	**P-selectin(ng/ml)**	44.0 (13.7–101.6)	44.0 (21.7–97.4)	43.7 (13.7–101.6)	0.991
	**ICAM-3 (ng/ml)**	1.0 (0.2–4.9)	0.9 (0.2–4.9)	1.1 (0.5–2.9)	0.459
	**TM (ng/ml)**	**12.9 (SD 3.03)**	**12.0 (SD 2.73)**	**13.5 (SD 2.75)**	0.041*
	**MMP-1 (ng/ml)**	31.0 (2–140)	28.0 (2–105)	31.5 (2–140)	0.779
	**MMP-3 (ng/ml)**	52.0 (11–255)	48.5 (11–255)	55 (11–133)	0.251
	**MMP-9 ng/ml)**	99.0 (22–389)	111.0 (34–267)	81.5 (22–389)	0.152
**Pro-inflammatory**	**CRP (μg/ml)**	5.1 (0.5–140.3)	2.7 (0.6–68.1)	5.8 (0.5–140.3)	0.094
	**SAA (μg/ml)**	9.0 (0.7–231.1)	9.4 (1.3–165.4)	8.1 (0.7–231.1)	0.694
	**IL6 (pg/ml)**	**1.9 (0.6–131.5)**	**1.3 (0.8–7.4)**	**2.7 (0.6–131.5)**	0.011*
	**IL8 (pg/ml)**	14.3 (3.5–185.7)	12.6 (4.5–31.9)	15.9 (3.5–185.7)	0.091
	**TNF-α (pg/ml)**	6.0 (3.7–15.9)	6.0 (3.7–12.5)	6.2 (3.8–15.9)	0.766
**Growth Factors**	**bFGF (pg/ml)**	5.0 (0–28)	5.0 (0–28)	5.5 (0–21)	0.925
	**PIGF (pg/ml)**	27 (14–60)	26 (14–45)	27 (18–60)	0.333
	**Flt-1 (pg/ml)**	253 (139–1434)	255 (155–396)	251 (139–1434)	0.427
	**VEGF (pg/ml)**	636.5 (239–1732)	673.0 (280–1361)	611.0 (239–1732)	0.791
	**Leptin (pg/ml)**	**7751 (247–169213)**	**18639.5 (432–169213)**	**6972 (247–162842)**	0.011*
	**Insulin (pg/ml)**	451 (0–3195)	499 (131–3195)	437 (0–2224)	0.500

bFGF = Basic Fibroblast Growth Factor, BMI = Body Mass Index, BP = Blood Pressure, cm = centimetre, CRP = C-Reactive Protein, DBP = Diastolic BP, ECW = Extracellular Water, FTI = Fat Tissue Index, Flt-1 = Soluble fms-like tyrosine kinase-1, g = gram, hr = hour, ICAM-1 = Intercellular Adhesion, Molecule-1, IL = Inteleukin, Kg = Killogram, L = Litre, LTI = Lean Tissue Index, m = meter, MAP = Mean Arterial Pressure, Median P50 = red blood cell width (in micrometer), ml = millilitres, mmHg = millimetres of mercury, MMP = Matrix Metalloproteinase, ng = nanogram, OH = Overhydration Index, PIGF = Placenta Growth Factor, PBR = Perfused Boundary Region (in micrometers), pg = picogram, PWV = Pulse Wave Velocity, s = second, SAA = Soluble Amyloid A, SBP = Systolic BP, Sig = Statistical Significance (p-value), TBW = Total Body Water, TM = Thrombomodulin, TNF = Tumour Necrosis Factor, VCAM-1 = Vascular Cell Adhesion Molecule-1, VEGF = Vascular Endothelial Growth Factor, μg = microgram.

* Highlights Result with statistical significance at the level of p<0.05.

### Predictor assessment for overhydration (OH/ECW>7%) using multivariate logistic regression

Following univariate analysis of potential predictors of OH/ECW>7% from participants’ demographic, HD parameters, CV and biomarker profiles (S5 Table), VCAM-1, TM, IL6 and Leptin were then sequentially entered in a multivariate model adjusted for age, DCS, weekly hours of prescribed dialysis FTI and LTI (Table 4). High serum soluble VCAM-1 (VCAM-1/10: OR 1.04, 95% CI 1.01–1.07, p = 0.01) with low levels of Leptin (OR 0.97, 95% CI 0.95–0.99, p = 0.01) were independently associated with overhydration in the best fit model (AUC = 0.816) (Table 4). TM and IL6 were excluded from the final model.

**Table 4:** Multivariate analysis of overhydration predictors.

Predictor	Univariate Analysis		Multivariate Model	
	OR (95% CI)	Sig	OR (95% CI)	Sig
**Age**	1.04 (1.00–1.07)	0.055	1.01 (0.98–1.07)	0.401
**FTI**	0.95 (0.87–1.04)	0.294	1.13 (0.95–1.34)	0.184
**LTI**	0.95(0.87–1.04)	0.294	1.09 (0.84–1.42)	0.530
**Davies Comorbidity Score**	**1.62 (1.06–2.48)**	0.026*	1.38 (0.74–2.57)	0.315
**Hours of HD per week**	0.99 (0.92–1.07)	0.823	0.94 (0.85–1.05)	0.260
VCAM-1/10**	**1.04 (1.01–1.06)**	0.003*	**1.04 (1.01–1.07)**	0.014*
**Thrombomodulin**	**1.19 (1.003–1.41)**	0.047*		
**IL6**	**1.42 (1.06–1.92)**	0.021*		
Leptin (ng/ml)***	**0.99 (0.98–0.998)**	0.016*	**0.97 (0.95–0.99)**	0.007*

CI = Confidence Interval, FTI = Fat tissue index, HD = Haemodialysis, IL = Interleukin, LTI = Lean Tissue Index, OR = Odd Ration, Sig = Statistical Significance, VCAM-1 = Vascular Cell Adhesion Molecule-1.

* Highlights Result with statistical significance at the level of p<0.05.

** OR for VCAM-1/10 indicates the risk of ovehydration for a rise in VCAM-1 by 10 ng/ml.

*** OR for Leptin indicates the risk of overhydration for a drop in Leptin levels by 1 ng/ml rather than pg/ml.

#### Assessing the relationship overhydration predictors with ECW/TBW using sequential additive multivariate linear regression model

To assess the strength of our findings and to avoid over adjustment for body composition by using parameters containing the absolute OH index we also used the bioratio of ECW/TBW to assess potential predictors of ECW water expansion. VCAM-1 showed a positive correlation to ECW/TBW (Beta = 0.306, 95% CI 0.07–0.05, p = 0.01). ECW/TBW was also associated with older age, comorbidity, FTI, LTI, Matrix Metalloproteinase (MMP)-1 and PBR thickness of the largest measured capillaries (20–25μm) (Table 5).

**Table 5:** Multivariate analysis of predictors of extracellular water expansion.

Cohort Characteristics		Univariate Analysis		Multivariate Analysis	
		Beta (95% CI)	Sig	Beta (95% CI)	Sig
**Age**		**0.503 (0.26–0.62)**	<0.001*	**0.410 (0.19–0.46)**	<0.001*
**Davies’ Comorbidity Score**		**0.468 (0.23–0.60)**	<0.001*	0.153 (-0.04–0.29)	0.134
**Hours of HD per week**		**-0.246 (-0.43–-0.01)**	0.037*	-0.045 (-0.18–0.10)	0.601
**FTI**		**0.351 (0.11–0.49)**	0.003*	**0.308 (0.11–0.41)**	0.001*
**LTI**		**-0.471 (-0.57–-0.22)**	<0.001*	**-0.245 (-0.35–-0.04)**	0.013*
**PWV**		0.165 (-0.08–0.43)	0.179		
**Capillarscopy**	**PBR 5–25**	0.131 (-0.10–0.33)	0.280		
	**PBR 5–9**	-0.043 (-0.26–0.18)	0.726		
	**PBR 10–19**	0.005 (-0.22–0.23)	0.970		
	**PBR 20–25**	**0.260 (0.02–0.44)**	0.030*		
	**Median P50**	-0.096 (-0.30–0.13)	0.427		
**VascularBiology**	**ICAM-1**	0.120 (-0.10–0.31)	0.315		
	**VCAM-1**	**0.302 (0.07–0.52)**	0.010*	**0.219 (0.03–0.36)**	0.019*
	**E-Selectin**	-0.201 (-0.39–0.03)	0.090		
	**P-Selectin**	0.013 (-0.21–0.24)	0.912		
	**ICAM-3**	0.009 (-0.01–0.01)	0.939		
	**Thrombomodulin**	0.065 (-0.18–0.31)	0.589		
	**MMP-1**	**0.270 (0.04–0.45)**	0.020*		
	**MMP-3**	-0.131 (-0.32–0.09)	0.272		
	**MMP-9**	-0.032 (-0.27–0.21)	0.792		
**Pro-inflammatory**	**CRP**	0.139 (-0.08–0.31)	0.244		
	**SAA**	0.107 (-0.12–0.32)	0.369		
	**IL6**	-0.016 (-0.21–0.18)	0.894		
	**IL8**	0.129 (-0.09–0.30)	0.279		
	**TNF-α**	0.031 (-0.18–0.23)	0.797		
**Growth Factors**	**bFGF**	0.085 (-0.14–0.29)	0.477		
	**PIGF**	0.114 (-0.11–0.30)	0.341		
	**Flt-1**	-0.030 (-0.23–0.18)	0.801		
	**VEGF**	0.064 (-0.15–0.27)	0.591		
	**Leptin**	0.131 (-0.10–0.34)	0.274		
	**Insulin**	-0.025 (-0.22–0.18)	0.833		

bFGF = Basic Fibroblast Growth Factor, CRP = C-Reactive Protein, FTI = Fat Tissue Index, Flt-1 = Soluble fms-like tyrosine kinase-1, HD = haemodialysis, hr = hour, ICAM-1 = Intercellular Adhesion, Molecule-1, IL = Interleukin, Median P50 = red blood cell width, MMP = Matrix Metalloproteinase, PIGF = Placenta Growth Factor, PBR = Perfused Boundary Region, PWV = Pulse Wave Velocity, SAA = Soluble Amyloid A, Sig = Statistical Significance (p-value), TNF = Tumour Necrosis Factor, VCAM-1 = Vascular Cell Adhesion Molecule-1, VEGF = Vascular Endothelial Growth Factor.

* Highlights Result with statistical significance at the level of p<0.05.

Significant predictors of OH/ECW status of >7% and ECW/TBW were added sequentially to a multivariate model. VCAM-1 significantly predicted ECW/TBW independent of any other variable (Beta = 0.219, p = 0.02) and adjusted for age, DCS and prescribed HD weekly hours in the model of best fit (R^2^ = 0.591, p>0.01). High FTI and low LTI were also associated with ECW/TBW in the same model. MMP-1, TM, IL6, Leptin and PBR (20–25 μm) were excluded from the final model.

## Discussion

Our results demonstrate a significant association of extracellular overhydration with endothelial and microinflammation biomarkers in HD patients. This might reflect an interaction between ECW expansion and vascular damage at the endothelial level. Although no relation was observed between macrocirculation and overhydration, and only minor associations were seen with measured microvascular parameters in vivo, VCAM-1, MMP-1, IL-6 and TM all correlated with either OH/ECW, ECW/TBW, or both.

TM is a trans-membrane glycoprotein expressed on the surface of endothelial cells and has both an anticoagulant and anti-inflammatory effect by binding thrombin and activating protein C [29,30]. Serum TM is released due to glycocalyx injury and is also a uremic retention product [29,30].

VCAM-1, the strongest predictor of overhydration in our cohort, is an adhesion molecule expressed on the vascular endothelium following neutrophil activation [31]. High levels of VCAM-1 have been reported in CKD and linked to the development of CV disease [32,33]. To our knowledge this is the first time VCAM-1 has been linked to hydration status. Neutrophil binding to adhesion molecules, that also include ICAM and E- and P- selectin, is mediated through integrins [34]. This process enables the migration and translocation of neutrophils across the intercellular junction, by altering the cellular endothelial cytoskeleton and increases vascular permeability [31,34,35]. The interaction of neutrophils with VCAM-1, is also thought to jeopardise the integrity of the endothelium further by activating transcellular permeability pathways [35] and through proteinase enzyme secretion from inflammatory that also alter the endothelial glycocalyx [34].

MMPs are such enzymes and act as key modulators of endothelial remodelling. They are secreted by endothelial cells, monocytes, neutrophils and vascular smooth muscle cells and activated in the presence of inflammatory cytokines [36]. While the involvement of MMPs in the process of atherosclerosis is clear, their direct role in the different pathophysiological pathways implicated is complex and findings to date are difficult to interpret [37]. MMP-1 is a collagenase and its role includes endothelial glycocalyx degradation and cleavage of adhesion molecules and surface factors from the endothelium [37]. This would explain why in our cohort MMP-1 levels were not independent from serum VCAM-1 levels. MMP-1 expression is variable and can be influenced by a wide range of inflammatory mediators, growth factors, cytokines and adhesion molecules but also states of oxidative stress [38,39].

One of the key cytokines implicated in the micro-inflammation related to states of oxidative stress, such as CKD, is IL-6 [40,41]. IL-6 is expressed in response to both inflammatory and stress-related stimuli by a number of cells including endothelial cells, T-lymphocytes, macrophages and fibroblasts [42]. One of its roles is to promote inflammatory cell proliferation and trans-endothelial migration [41] and is thought to be a more representative marker of microinflammation than CRP in CKD [40]. IL-6 has been linked with CV disease [43] and, together with VCAM-1, has been implicated in in-vitro and animal models of endothelial dysfunction in uraemia [44]. Recently Ioannou et al. described the association of VCAM-1 and IL-6 with LVMI in pre-dialysis CKD [45]. They also showed that the increase in LVMI correlated with increase in diuretic dose [45].

Another biomarker that showed an interesting association with overhydration in our study is Leptin. Adipose tissue, a major endocrine tissue and key contributor to inflammation [46], releases cytokines such as ILs and Leptin, predominantly in response to hypoxia and oxidative stress [47,48]. Best known for being an appetite suppressant that regulates body weight and energy balance, Leptin is also a regulator of vascular inflammation [48] and a retention product in CKD [46,49,50]. Leptin levels are often difficult to interpret as they are closely linked to both low LTI and high FTI [47,49]. This phenomenon might be what influences our finding. Both of these parameters were independently linked with ECW/TBW in our cohort. Whether Leptin levels reflect purely nutritional state or not, the nature of the interaction between body composition and ECW is unclear and might extend beyond the amount of salt and water intake [49]. There is also a debate whether some parameters used to calculate overhydration might lead to overestimation while other to underestimation based on body composition differences [51]. This might be the reason why we see slightly different associations between the two parameters of extracellular ovehydration used and the measured CV parameters in our study.

These observations, in conjunction with our findings, would support a relation between overhydration and endothelial injury. It is generally believed that overhydration is likely to promote vascular injury and endothelial dysfunction. The association of overhydration to systemic inflammation in the context of microvascular injury might suggest another dimension to the relationship between states of extracellular hydration excess and the vascular endothelium. The expansion of ECW across the vascular and interstitial compartments is more clearly elicited at the microvascular level. Vascular integrity at this levels and regulation of its barrier permeability may be involved in the development of overhydration and oedema but also the transcompartmental fluid movements during ultrafiltration (UF) [52]. The endothelial glycocalyx provides both a physical and an electrical barrier to fluid and particle translocation from the intravascular (IV) to the extravascular compartment [34]. Even when damaged the negatively charged proteoglycan coating the luminal surface of the endothelium will retain most its barrier properties to large particles such as cells and proteins, it will however, become extremely leaky to water [34].

The PBR of the endothelial glycocalyx of sublingual capillaries has been viewed as a measure of such injury to the glycocalyx. The PBR is the layer of the proteoglycan matrix that can be permeated by RBC. Thickening of this layer is associated with thinning of the impermeable layer of the glycocalyx and indicates endothelial dysfunction. Increased PBR thickness has been described in CKD [9] and HD [19] while in transplant recipients its size is similar to that of healthy volunteers [9]. UF during HD has also been implicated in reduced perfusion of the sublingual capillaries using a different capillaroscopy software to the one used in our study [27]. The microvascular endothelial activation has been reported to be independent from macrovascular parameters such as arterial stiffness [53]. Our study findings only confirm a weak association between PBR thickness and ECW/TBW limited to the larger measured capillaries. The association did not persist through the multivariate analysis perhaps due to the visualised alteration to the glycocalyx being mediated by the dominant circulating pro-inflammatory and endothelial factors.

Although the concept of endothelial injury promoting the development of overhydration in uraemia remains purely hypothetical, it’s a notion that is not unreasonable. The dynamic interaction between IV and interstitial fluid across the vascular endothelial barrier remains beyond the scope of our study but the importance of glycocalyx injury on compartmental fluid distribution warrants further investigation.

Our findings also confirm the association between systolic hypertension and overhydration and are in keeping with the published literature on the effects of hydration and interdialytic fluid gain on BP in HD patients [54–57]. We did not, however, find any association between overhydration and PWV. Bearing in mind that our cohort had an overall acceptable level of hydration, it is likely that at these levels of overhydration, other factors exert more influence on PWV than variations in ECW volume. Also the association of PWV and overhydration is not entirely clear-cut. Akdam et al. showed that PWV positively correlated to both visit BP and bioimpedance derived OH in a mixed group of participants with CKD (stages 3–5 and 5dPD), although only BP maintained this association following model adjustment [2]. Bia et al. were able to show that PWV correlated to both OH and OH/ECW [58] in an HD cohort similar to the one in our study, however their analysis was only adjusted for BP and not for other confounders such as age and comorbidity. Other studies showed that PWV fluctuates during the HD cycle, with values following HD treatment correlating to UF rate [59], and the highest measurements occurring on the third interdialytic day [60]. They inferred that fluid excess was responsible for these observations.

Our analysis has certain limitations. The study is analysing the relation of body hydration to CV measurements and biomarkers profiles during a single time point of steady state. Longitudinal follow up of these variables may shed further light, although such a design would have to overcome the overlap of confounders of both long term CV and hydration shifts. Also, our study population consist of a fairly heterogeneous and diverse group in both their demographic and treatment characteristics. To account for these differences we adjusted our analysis for major differences in these parameters. In addition to this the production of hormones such as Leptin could vary though the day and, as such, there might be a degree of sampling bias. However, the biomarkers analysed in the study are large sized molecules that are unlikely to be influenced by dialytic clearance.

## Conclusion

The study describes a relationship between microinflammation, endothelial dysfunction and extracellular hydration states in end stage kidney disease. Although overhydration and ECW expansion are likely to promote endothelial injury it is conceivable that compromise to the microvascular endothelium could also mediate the transposition of a protein-rich exudate into the IF altering the traditional Starling forces [11] that govern transcapillary fluid movement. The potential impact of non-hydrostatic factors (vascular microinflammation and endothelial dysfunction) on the capillary barrier and its permeability to both fluid and proteins need to be explored further in developing fluid models in uraemia.

## Supporting information

S1 Table Extended haemodialysis cohort demographic and dialysis profiles. (DOCX)

S2 Table Hydration and cardiovascular profiles of the participants on extended haemodialysis prescriptions stratified by their overhydration status. (DOCX)

S3 Table Conventional haemodialysis cohort demographic and dialysis profiles. (DOCX)

S4 Table Hydration and cardiovascular profiles of the participants on conventional haemodialysis prescriptions stratified by their overhydration status. (DOCX)

S5 Table Assessment of potential predictors of overhydration (OH/ECW >7%). (DOCX)

## References

[1] Mitsides N, Keane DF, Lindley E, Mitra S. Technology innovation for patients with kidney disease. *J Med Eng Technol*. 2014;39(7):424–433. doi: 10.3109/03091902.2015.1088089 26453039 10.3109/03091902.2015.1088089

[2] Akdam H, Öğünç H, Alp A, Ozbek O, Omurlu IK, Yenicerloglu Y et al Assessment of volume status and arterial stiffness in chronic kidney disease. *Ren Fail*. 2014;36(1):28–34. doi: 10.3109/0886022X.2013.830224 24028203 10.3109/0886022X.2013.830224

[3] Lin Y-P, Chen C-H, Yu W-C, Hsu T-L, Ding PY-A, Yang W-C. Left ventricular mass and hemodynamic overload in normotensive hemodialysis patients. *Kidney Int*. 2002;62(5):1828–1838. doi: 10.1046/j.1523-1755.2002.00610.x 12371986 10.1046/j.1523-1755.2002.00610.x

[4] Tsai Y-CC, Chiu Y-WW, Tsai J-CC, Kuo H-TT, Hung C-CC, Hwang S-JJ, et al Association of fluid overload with cardiovascular morbidity and all-cause mortality in stages 4 and 5 CKD. *Clin J Am Soc Nephrol*. 2015;10(1):39–46. doi: 10.2215/CJN.03610414 25512646 10.2215/CJN.03610414 PMC4284411

[5] Hung S-C, Kuo K-L, Peng C-H, Wu C-H, Wang Y-C, Tang D-C. Volume overload correlates with cardiovascular risk factors in patients with chronic kidney disease. *Kidney Int*. 2014;85(3):703–709. doi: 10.1038/ki.2013.336 24025647 10.1038/ki.2013.336

[6] Meijers BKI, Claes K, Bammens B, de Loor H, Viaene L, Verbeke K, et al p-Cresol and cardiovascular risk in mild-to-moderate kidney disease. *Clin J Am Soc Nephrol*. 2010;5(7):1182–1189. doi: 10.2215/CJN.07971109 20430946 10.2215/CJN.07971109 PMC2893077

[7] Meijers BKI, Bammens B, De Moor B, Verbeke K, Vanrenterghem Y, Evenepoel P. Free p-cresol is associated with cardiovascular disease in hemodialysis patients. *Kidney Int*. 2008;73(10):1174–1180. doi: 10.1038/ki.2008.31 18305466 10.1038/ki.2008.31

[8] Beckman JA, Creager MA, Libby P, Carr ME, Turner RC, Plutzky J, et al Diabetes and Atherosclerosis. *JAMA*. 2002;287(19):2570 doi: 10.1001/jama.287.19.2570 12020339 10.1001/jama.287.19.2570

[9] Dane MJC, Khairoun M, Lee DH, vadn den Berg BM, Eskens BJM, Boels MGS, et al Association of kidney function with changes in the endothelial surface layer. *Clin J Am Soc Nephrol*. 2014;9(4):698–704. doi: 10.2215/CJN.08160813 24458084 10.2215/CJN.08160813 PMC3974363

[10] Oberleithner H. Vascular endothelium: a vulnerable transit zone for merciless sodium. *Nephrol Dial Transplant*. 2014;29(2):240–246. doi: 10.1093/ndt/gft461 24335504 10.1093/ndt/gft461

[11] Levick JR, Michel CC. Microvascular fluid exchange and the revised Starling principle. *Cardiovasc Res*. 2010;87(2):198–210. doi: 10.1093/cvr/cvq062 20200043 10.1093/cvr/cvq062

[12] Machnik A, Dahlmann A, Kopp C, Goss J, Wagner H, van Rooijen N, et al Mononuclear phagocyte system depletion blocks interstitial tonicity-responsive enhancer binding protein/vascular endothelial growth factor C expression and induces salt-sensitive hypertension in rats. *Hypertension*. 2010;55(3):755–761. doi: 10.1161/HYPERTENSIONAHA.109.143339 20142563 10.1161/HYPERTENSIONAHA.109.143339

[13] Dekker MJE, Marcelli D, Canaud BJ, Carioni P, Wang Y, Grassmann A, et al Impact of fluid status and inflammation and their interaction on survival: a study in an international hemodialysis patient cohort. Kidney Int. 2017;91(5):1214–1223 doi: 10.1016/j.kint.2016.12.008 28209335 10.1016/j.kint.2016.12.008

[14] Davies SJ, Russell L, Bryan J, Phillips L, Russell GI. Comorbidity, urea kinetics, and appetite in continuous ambulatory peritoneal dialysis patients: their interrelationship and prediction of survival. *Am J Kidney Dis*. 1995;26(2):353–361. 7645541 10.1016/0272-6386(95)90657-6

[15] Chamney PW, Wabel P, Moissl UM, Muller MJ, Bosy-Westphel A, Korth O, et al A whole-body model to distinguish excess fluid from the hydration of major body tissues. *Am J Clin Nutr*. 2007;85(1):80–89. 17209181 10.1093/ajcn/85.1.80

[16] Wizemann V, Wabel P, Chamney P, Zaluska W, Moissl U, Rode C, et al The mortality risk of overhydration in haemodialysis patients. *Nephrol Dial Transplant*. 2009;24(5):1574–1579. doi: 10.1093/ndt/gfn707 19131355 10.1093/ndt/gfn707 PMC2668965

[17] Davies SJ, Davenport A. The role of bioimpedance and biomarkers in helping to aid clinical decision-making of volume assessments in dialysis patients. *Kidney Int*. 2014;86(3):489–496. doi: 10.1038/ki.2014.207 24918155 10.1038/ki.2014.207

[18] Jiang B, Liu B, McNeill KL, Chowienczyk PJ. Measurement of Pulse Wave Velocity Using Pulse Wave Doppler Ultrasound: Comparison with Arterial Tonometry. *Ultrasound Med Biol*. 2008;34(3):509–512. doi: 10.1016/j.ultrasmedbio.2007.09.008 18031922 10.1016/j.ultrasmedbio.2007.09.008

[19] Vlahu CA, Lemkes BA, Struijk DG, Koopman MG, Krediet RT, Vink H. Damage of the endothelial glycocalyx in dialysis patients. *J Am Soc Nephrol*. 2012;23(11):1900–1908. doi: 10.1681/ASN.2011121181 23085635 10.1681/ASN.2011121181 PMC3482728

[20] VanTeeffelen JW, Brands J, Stroes ES, Vink H. Endothelial Glycocalyx: Sweet Shield of Blood Vessels. *Trends Cardiovasc Med*. 2007;17(3):101–105. doi: 10.1016/j.tcm.2007.02.002 17418372 10.1016/j.tcm.2007.02.002

[21] Edul VSK, Enrico C, Laviolle B, Vazquez AR, Ince C, Dubin A. Quantitative assessment of the microcirculation in healthy volunteers and in patients with septic shock*. *Crit Care Med*. 2012;40(5):1443–1448. doi: 10.1097/CCM.0b013e31823dae59 22430243 10.1097/CCM.0b013e31823dae59

[22] VanTeeffelen JWGE. How to prevent leaky vessels during reperfusion? Just keep that glycocalyx sealant in place! *Crit Care*. 2008;12(4):167 doi: 10.1186/cc6939 18638363 10.1186/cc6939 PMC2575556

[23] Lee DH, Dane MJC, Van Den Berg BM, Boels MGS, Van Teeffelen JW, De Mutsert R, et al Deeper penetration of erythrocytes into the endothelial glycocalyx is associated with impaired microvascular perfusion. *PLoS One*. 2014;9(5):1–8. doi: 10.1371/journal.pone.0096477 24816787 10.1371/journal.pone.0096477 PMC4015985

[24] Gu YM, Wang S, Zhang L, Liu YP, Thijs L, Petit T, et al Characteristics and determinants of the sublingual microcirculation in populations of different ethnicity. *Hypertension*. 2015;65(5):993–1001. doi: 10.1161/HYPERTENSIONAHA.114.05119 25712718 10.1161/HYPERTENSIONAHA.114.05119

[25] Takahashi W, Watanabe E, Fujimura L, Watanabe-Takano H, Yoshidime H, swanson PE et al Kinetics and protective role of autophagy in a mouse cecal ligation and puncture-induced sepsis. *Crit Care*. 2013;17(4):1 doi: 10.1186/cc12839 23883625 10.1186/cc12839 PMC4056358

[26] Donati A, Damiani E, Domizi R, Romano R, Adriano E, Pelaia P, et al Alteration of the sublingual microvascular glycocalyx in critically ill patients. *Microvasc Res*. 2013;90:86–89. doi: 10.1016/j.mvr.2013.08.007 23988876 10.1016/j.mvr.2013.08.007

[27] Bemelmans RHH, Boerma EC, Barendregt J, Ince C, Rommes JH, Spronk PE. Changes in the volume status of haemodialysis patients are reflected in sublingual microvascular perfusion. *Nephrol Dial Transplant*. 2009;24(11):3487–3492. doi: 10.1093/ndt/gfp267 19515801 10.1093/ndt/gfp267

[28] Cornelis T, Broers NJH, Titulaer DCLM, Henskens YM, van Oerle R, van der Sande FM, et al Effects of Ultrapure Hemodialysis and Low Molecular Weight Heparin on the Endothelial Surface Layer. *Blood Purif*. 2014;38(3–4):203–210. doi: 10.1159/000369055 25531879 10.1159/000369055

[29] Bao Y-S, Jia X-B, Wang D, Liu R-C, Zou C-B, Na S-P. Characterization of soluble thrombomodulin levels in patients with stage 3–5 chronic kidney disease. *Biomarkers*. 2014;19(4). doi: 10.3109/1354750X.2014.904000 24854597 10.3109/1354750X.2014.904000

[30] Zahran M, Nasr FM, Metwaly AA, El-Sheikh N, Khalil NSA, Harba T. The Role of Hemostatic Factors in Atherosclerosis in Patients with Chronic Renal Disease. *Electron physician*. 2015;7(5):1270–1276. doi: 10.14661/1270 26435827 10.14661/1270 PMC4590563

[31] van Wetering S, van den Berk N, van Buul JD, Mul J FP, Lommerse I, Mous R, et al VCAM-1-mediated Rac signaling controls endothelial cell-cell contacts and leukocyte transmigration. *Am J Physiol Cell Physiol*. 2003;(285):C343–C352. 12700137 10.1152/ajpcell.00048.2003

[32] Caballo C, Palomo M, Cases A, Galan AN, Molina P, Vera M, et al NFκB in the development of endothelial activation and damage in uremia: an in vitro approach. *PLoS One*. 2012;7(8):e43374 doi: 10.1371/journal.pone.0043374 22937042 10.1371/journal.pone.0043374 PMC3425561

[33] Patel T V, Mittal B V, Keithi-Reddy SR, Duffield JS, Singh AK. Endothelial activation markers in anemic non-dialysis chronic kidney disease patients. *Nephron Clin Pract*. 2008;110(4):c244–50. doi: 10.1159/000167872 18974656 10.1159/000167872 PMC2818376

[34] Rodrigues SF, Granger DN. Blood cells and endothelial barrier function. *Tissue Barriers*. 2015;3(1–2):e978720 doi: 10.4161/21688370.2014.978720 25838983 10.4161/21688370.2014.978720 PMC4372023

[35] Distasi MR, Ley K. Opening the flood-gates: how neutrophil-endothelial interactions regulate permeability. doi: 10.1016/j.it.2009.07.012 19783480 10.1016/j.it.2009.07.012 PMC2767453

[36] Nugent WH, Mishra N, Strauss JF 3rd, Walsh SW. Matrix Metalloproteinase 1 Causes Vasoconstriction and Enhances Vessel Reactivity to Angiotensin II via Protease-Activated Receptor 1. *Reprod Sci*. 2016;23(4):542–548. doi: 10.1177/1933719115607998 26438597 10.1177/1933719115607998 PMC5933188

[37] Friese RS, Rao F, Khandrika S, Thomas B, Ziegler MG, Schmid-Schonbein GW, et al Matrix Metalloproteinases: Discrete Elevations in Essential Hypertension and Hypertensive End-Stage Renal Disease. *Clin Exp Hypertens*. 2009;31(7):521–533. doi: 10.3109/10641960802668730 19886850 10.3109/10641960802668730 PMC2875383

[38] Death AK, Fisher EJ, McGrath KCY, Yue DK. High glucose alters matrix metalloproteinase expression in two key vascular cells: Potential impact on atherosclerosis in diabetes. *Atherosclerosis*. 2003;168(2):263–269. doi: 10.1016/S0021-9150(03)00140-0 12801609 10.1016/s0021-9150(03)00140-0

[39] Nagase H, Woessner JF. Matrix Metalloproteinases. *J Biol Chem*. 1999;274(31):21491–21494. 10419448 10.1074/jbc.274.31.21491

[40] Lee BT, Ahmed FA, Hamm LL, Teran FJ, Chen C-S, Liu Y, et al Association of C-reactive protein, tumor necrosis factor-alpha, and interleukin-6 with chronic kidney disease. *BMC Nephrol*. 2015;16(1):77 doi: 10.1186/s12882-015-0068-7 26025192 10.1186/s12882-015-0068-7 PMC4449580

[41] Bijuklic K, Jennings P, Kountchev J, Hasslacher J, Aydin S, Sturn D, et al Migration of leukocytes across an endothelium-epithelium bilayer as a model of renal interstitial inflammation. *Am J Physiol—Cell Physiol*. 2007;293(1). 10.1152/ajpcell.00419.2006 17428840

[42] Waage A, Slupphaug G, Shalaby R. Glucocorticoids inhibit the production of IL 6 from monocytes, endothelial cells and fibroblasts. *Eur J Immunol*. 1990;20(11):2439–2443. doi: 10.1002/eji.1830201112 2253684 10.1002/eji.1830201112

[43] Spoto B, Mattace-Raso F, Sijbrands E, Leonardis D, Testa A, Pisano A, et al Association of IL-6 and a functional polymorphism in the IL-6 gene with cardiovascular events in patients with CKD. *Clin J Am Soc Nephrol*. 2015;10(2):232–240. doi: 10.2215/CJN.07000714 25492254 10.2215/CJN.07000714 PMC4317745

[44] D ‘Apolito M, Du X, Pisanelli D, Pettoello-Montovani M, Campanozzi A, Giacco F, et al Urea-induced ROS cause endothelial dysfunction in chronic renal failure HHS Public Access. *Atherosclerosis*. 2015;239(2):393–400. doi: 10.1016/j.atherosclerosis.2015.01.034 25682038 10.1016/j.atherosclerosis.2015.01.034 PMC4361277

[45] Ioannou K, Stel VS, Dounousi E, Jager KT, Papagianni A, Pappas K, et al Inflammation, endothelial dysfunction and increased left ventricular mass in chronic kidney disease (CKD) patients: A longitudinal study. Passino C, ed. *PLoS One*. 2015;10(9):e0138461 doi: 10.1371/journal.pone.0138461 26398099 10.1371/journal.pone.0138461 PMC4580570

[46] Bernardo AP, Fonseca I, Oliveira JC, Santos O, Carvalho MJ, Cabrita A, et al Adipokines in Peritoneal Dialysis: Relevant Clinical Impact According to Body Composition. *Ther Apher Dial*. 2015;19(2):144–153. doi: 10.1111/1744-9987.12239 25363550 10.1111/1744-9987.12239

[47] Van de Voorde J, Pauwels B, Boydens C, Decaluwé K. Adipocytokines in relation to cardiovascular disease. *Metabolism*. 2013;62(11):1513–1521. doi: 10.1016/j.metabol.2013.06.004 23866981 10.1016/j.metabol.2013.06.004

[48] Kougias P, Chai H, Lin PH, Yao Q, Lumsden AB, Chen C. Effects of Adipocyte-Derived Cytokines on Endothelial Functions: Implication of Vascular Disease. *J Surg Res*. 2005;126(1):121–129. doi: 10.1016/j.jss.2004.12.023 15916985 10.1016/j.jss.2004.12.023

[49] Ahbap E, Sakaci T, Kara E, Sahutoglu T, Koc Y, Basturk M, et al Relationship between relative interdialytic weight gain and serum leptin levels, nutrition, and inflammation in chronic hemodialysis patients. *Clin Nephrol*. 2015;83(3):154–160. 25685870 10.5414/cn108450

[50] Beberashvili I, Sinuani I, Azar A, Yasur H, Feldman L, Averbukh Z, et al Longitudinal study of leptin levels in chronic hemodialysis patients. *Nutr J*. 2011;10:68 doi: 10.1186/1475-2891-10-68 21676262 10.1186/1475-2891-10-68 PMC3132708

[51] Lindley EJ, Lopot F. The use of bioimpedance to aid volume assessment in dialysis patients. *Kidney Int*. 2015;87(1):240 doi: 10.1038/ki.2014.310 25549124 10.1038/ki.2014.310

[52] Mitra S. Extracellular hydration, cardiovascular risk, and the interstitium: a three-dimensional view. *Kidney Int*. 2014;85(3):510–512. doi: 10.1038/ki.2013.481 24583986 10.1038/ki.2013.481

[53] van Sloten TT, Czernichow S, Houben AJ, Protogerou AD, Henry RM, Muris DM, et al Association Between Arterial Stiffness and Skin Microvascular Function: The SUVIMAX2 Study and The Maastricht Study. *Am J Hypertens*. 2015;28(7):868–876. doi: 10.1093/ajh/hpu246 25523296 10.1093/ajh/hpu246

[54] Nongnuch A, Campbell N, Stern E, El-Kateb S, Fuentes L, Davenport A. Increased postdialysis systolic blood pressure is associated with extracellular overhydration in hemodialysis outpatients. *Kidney Int*. 2014;87(2):1–6. doi: 10.1038/ki.2014.276 25075771 10.1038/ki.2014.276

[55] Chen Y-C, Lin C-J, Wu C-J, Chen H-H, Yeh J-C. Comparison of extracellular volume and blood pressure in hemodialysis and peritoneal dialysis patients. *Nephron Clin Pract*. 2009;113(2):c112–6. doi: 10.1159/000228543 19602907 10.1159/000228543

[56] Katzarski K, Charra B, Luik A, Nisell J. Fluid state and blood pressure control in patients treated with long and short haemodialysis. *Nephrol Dial Transplant*. 1999:369–375. 10069191 10.1093/ndt/14.2.369

[57] Kim ED, Sozio SM, Estrella MM, Jaar BG, Shafi T, Meoni LA, et al Cross-sectional association of volume, blood pressures, and aortic stiffness with left ventricular mass in incident hemodialysis patients: the Predictors of Arrhythmic and Cardiovascular Risk in End-Stage Renal Disease (PACE) study. *BMC Nephrol*. 2015;16:131 doi: 10.1186/s12882-015-0131-4 26249016 10.1186/s12882-015-0131-4 PMC4528691

[58] Bia D, Galli C, Valtuille R, Zocalo Y, Wray SA, Armentano RL, et al Hydration Status Is Associated with Aortic Stiffness, but Not with Peripheral Arterial Stiffness, in Chronically Hemodialysed Patients. *Int J Nephrol*. 2015;2015 doi: 10.1155/2015/628654 26167301 10.1155/2015/628654 PMC4488172

[59] Di Iorio B, Nazzaro P, Cucciniello E, Bellizzi V. Influence of haemodialysis on variability of pulse wave velocity in chronic haemodialysis patients. *Nephrol Dial Transplant*. 2010;25(5):1579–1583. doi: 10.1093/ndt/gfp662 20031931 10.1093/ndt/gfp662

[60] Koutroumbas G, Georgianos PI, Sarafidis PA, Protogerou A, Karpetas A, Vaklanis P, et al Ambulatory aortic blood pressure, wave reflections and pulse wave velocity are elevated during the third in comparison to the second interdialytic day of the long interval in chronic haemodialysis patients. *Nephrol Dial Transplant*. 2015;30(12):2046–2053. doi: 10.1093/ndt/gfv090 25920919 10.1093/ndt/gfv090 PMC4832990

