# Supplementary material for: Extracellular overhydration linked with endothelial dysfunction in the context of inflammation in haemodialysis dependent chronic kidney disease
Source: PLoS One. 2017 Aug 22;12(8):e0183281. doi: 10.1371/journal.pone.0183281 (PMC5568741; doi:10.1371/journal.pone.0183281)
Supplement: S1 Table — (DOCX) [file pone.0183281.s001.docx]

| Extended HD Cohort  Characteristics | | Entire Cohort n=36 | No overhydration (OH/ECW < 7%) n=17 | Overhydration (OH/ECW > 7%) n=19 | Sig |
| --- | --- | --- | --- | --- | --- |
| Age (year) | | 52.6 (SD 13.5) | 48.7 (SD 13.0) | 56.1 (SD 13.3) | 0.100 |
| Sex: Male | | 27 (75%) | 12 (70.6%) | 15 (78.9%) | 0.563 |
| Ethnicity | **White** | 29 (80.6%) | 14 (82.4%) | 15 (78.9%) | 0.546 |
|  | **Black** | 1 (2.8%) | - | 1 (5.3%) |  |
|  | **Asian** | 5 (13.9%) | 3 (17.6%) | 2 (10.5%) |  |
|  | **Other** | 1 (2.8%) | - | 1 (5.3%) |  |
| Dialysis Vintage (months) | | 84.0 (10-432) | 72 (10-276) | 96 (14-432) | 0.154 |
| Residual Urine Output | | 3 (8.3%) | 0 (0%) | 3 (15.8%) | 0.087 |
| Previous Transplant | | 19 (52.8 %) | 8 (47.1%) | 11 (57.9%) | 0.516 |
| Diabetes Mellitus | | 9 (25%) | 4 (23.5%) | 5 (26.3%) | 0.847 |
| CVD | | **6 (16.7%)** | **0 (0%)** | **6 (31.6%)** | **0.011*** |
| Smoking | | 2 (5.6%) | 2 (11.8%) | 0 (0 %) | 0.124 |
| Davies Comorbidity Score | | 1 (0-4) | 1 (0-3) | 2 (0-4) | 0.089 |
| Number of BP medication | | 1 (0-4) | 1 (0-2) | 1 (0-4) | 0.187 |
| HD Parameters | **Hrs per wk** | 17.5 (13.5-42.5) | 16.5 (13.5-42.5) | 20 (14-32) | 0.264 |
|  | **HD frequency per wk** | 3.5 (3-6) | 3.5 (3-5) | 3.5 (3-6) | 0.841 |
|  | **HD session length** | 5 (2.5-9.0) | 5 (4-8.5) | 5 (2.5-9) | 0.283 |
|  | **Standard Kt/v (n=67)** | 2.55 (SD 0.45) | 2.57 (SD 0.48) | 2.55 (SD 0.44) | 0.836 |

**S1 Table. Extended Haemodialysis Cohort Demographic and Dialysis Profiles**. BP= Blood Pressure, CVD= Cardiovascular Disease, ECW= Extracellular Water, HD= Haemodialysis, hr= hour, Kg= Kilogram, OH= Overhdration Index, SD= Standard Deviation, Sig= Statistical Significance (p-value). ***** Highlights Result with statistical significance at the level of p<0.05.
