# Supplementary material for: Extracellular overhydration linked with endothelial dysfunction in the context of inflammation in haemodialysis dependent chronic kidney disease
Source: PLoS One. 2017 Aug 22;12(8):e0183281. doi: 10.1371/journal.pone.0183281 (PMC5568741; doi:10.1371/journal.pone.0183281)
Supplement: S2 Table — (DOCX) [file pone.0183281.s002.docx]

| Fluid and  Cardiovascular Parameters | | | Extended HD Cohort  N=36 | No overhydration  (OH/ECW < 7%) n=17 | Overhydration  (OH/ECW > 7%) n=19 | Sig |
| --- | --- | --- | --- | --- | --- | --- |
| Body Composition | **OH (L)** | | **1.4 (-1.8-5.7)** | **-0.2 (-1.8-1.3)** | **2.2 (1.1-5.7)** | **<0.001*** |
|  | **OH/ECW (%)** | | 6.10 (SD 9.05) | -1.8 (SD 5.7) | 13.2 (SD 4.2) | <0.001* |
|  | **TBW (L)** | | 37.64 (SD 8.62) | 37.8 (SD 10.0) | 37.5 (SD 7.5) | 0.917 |
|  | **ECW/TBW** | | **0.48 (SD 0.03)** | **0.46 (SD 0.03)** | **0.50 (SD 0.03)** | **<0.001*** |
|  | **Weight (Kg)** | | 82.0 (SD 17.2) | 83.9 (SD 17.4) | 80.2 (SD 17.3) | 0.525 |
|  | **BMI (Kg/m2)** | | 27.4 (SD 5.1) | 28.1 (SD 5.8) | 26.7 (SD 4.4) | 0.424 |
|  | **LTI (Kg/m2)** | | 12.4 (SD 2.5) | 12.7 (SD 2.5) | 12.2 (SD 2.7) | 0.554 |
|  | **FTI (Kg/m2)** | | 13.9 (5.1-35.0) | 14.4 (9.2-35.0) | 13.5 (5.1-27.2) | 0.320 |
| Visit BP (mmHg) | **Systolic** | | 137.0 (SD 29.7) | 128.8 (SD 24.7) | 144.4 (SD 32.4) | 0.116 |
|  | **Diastolic** | | 75.3 (SD 14.8) | 74.9 (SD 15.7) | 75.5 (SD 14.3) | 0.908 |
|  | **MAP** | | 116.4 (SD 23.8) | 110.8 (SD 21.0) | 121.4 (SD 25.6) | 0.186 |
| PWV (m/s) (n=17:19) | | | 8.38 (SD 2.58) | 8.1 (SD 2.35) | 8.7 (SD 2.81) | 0.516 |
| 24hr BP (mmHg) (n=15:16) | **Systolic BP** | | **134.3 (SD 26.5)** | **124.3 (SD 21.6)** | **143.8 (SD 27.8)** | **0.039*** |
|  | **Diastolic BP** | | 82.6 (SD 15.7) | 78.1 (SD 15.2) | 86.8 (SD 15.4) | 0.128 |
|  | **MAP** | | 99.8 (SD 18.6) | 93.5 (SD 16.7) | 105.8 (SD 18.8) | 0.066 |
| Capillaroscopy | **PBR 5-25** | | 1.93 (SD 0.27) | 1.96 (SD 0.25) | 1.97 (SD 0.28) | 0.411 |
|  | **PBR 5-9** | | 0.94 (0.79-1.11) | 0.97 (0.78-1.09) | 0.92 (0.79-1.11) | 0.267 |
|  | **PBR 10-19** | | 2.09 (SD 0.24) | 2.17 (SD 0.16) | 2.03 (SD 0.29) | 0.092 |
|  | **PBR 20-25** | | 2.50 (SD 0.59) | 2.50 (SD 0.45) | 2.50 (SD 0.70) | 0.991 |
|  | **Median P-50** | | 11.19 (SD 1.52) | 11.40 (SD 1.42) | 11.01 (SD 1.62) | 0.461 |
| Vascular Biology | **ICAM-1 (ng/ml)** | | 437.6 (SD 111.5) | 410.5 (SD 93.4) | 461.8 (SD 122.8) | 0.171 |
|  | **VCAM-1 (ng/ml)** | | **1108.1 (SD 276.8)** | **945.4 (SD 196.1)** | **1253.6 (SD 259.8)** | **<0.001*** |
|  | **E-selectin (ng/ml)** | | 10.9 (6.9-29.5) | 10.8 (6.9-29.5) | 11.0 (7.0-20.5) | 0.912 |
|  | **P-selectin (ng/ml)** | | 42.0 (13.7-97.4) | 43.9 (26.0-97.4) | 41.8 (13.7-70.4) | 0.419 |
|  | **ICAM-3 (ng/ml)** | | 1.0 (0.5-2.9) | 1.1 (0.7-2.2) | 1.0 (0.5-2.9) | 0.873 |
|  | **Thrombomodulin (ng/ml)** | | 13.2 (SD 2.83) | 12.4 (SD 3.03) | 13.9 (SD 2.49) | 0.104 |
|  | **MMP-1 (ng/ml)** | | 34.9 (SD 21.5) | 34.9 (SD 24.1) | 34.8 (SD 19.7) | 0.996 |
|  | **MMP-3 (ng/ml)** | | 62.2 (SD 35.7) | 57.9 (SD 36.2) | 66.0 (SD 35.9) | 0.510 |
|  | **MMP-9 (ng/ml)** | | 95.9 (SD 48.4) | 98.3 (SD 54.1) | 93.8 (SD 44.8) | 0.786 |
| Pro-inflammatory | **CRP (μg/ml)** | | 5.8 (0.5-96.0) | 2.8 (0.7-29.2) | 7.1 (0.5-96.0) | 0.235 |
|  | **SAA (μg/ml** | | 9.2 (0.7-231.1) | 9.3 (1.3-83.2) | 8.9 (0.7-231.1) | 0.912 |
|  | **IL6 (pg/ml)** | | **1.9 (0.8-131.5)** | **1.2 (0.8-4.1)** | **3.3 (0.9-131.5)** | **0.017*** |
|  | **IL8 (pg/ml)** | | 12.6 (3.5-62.0) | 10.6 (6.8-31.9) | 14.4 (3.5-62.0) | 0.145 |
|  | **TNF-α (pg/ml)** | | 6.2 (3.7-15.9) | 6.1 (3.7-12.5) | 6.2 (3.8-15.9) | 0.937 |
| Growth Factors | | **bFGF (pg/ml)** | 6.81 (SD 5.77) | 6.71 (SD 5.88) | 6.90 (SD 5.83) | 0.924 |
|  |  | **PIGF (pg/ml)** | 29.4 (SD 9.2) | 28.2 (SD 9.2) | 30.5 (SD 9.4) | 0.464 |
|  |  | **Flt-1 (pg/ml)** | 255 (139-369) | 256 (216-369) | 243 (139-318) | 0.516 |
|  |  | **VEGF (pg/ml)** | 732.1 (SD 299.5) | 778.2 (SD 356.9) | 690.8 (SD 239.6) | 0.402 |
|  |  | **Leptin (pg/ml)** | **7131 (247-167893)** | **114584 (1049-167893)** | **3852 (247-126013)** | **0.014*** |
|  |  | **Insulin (pg/ml)** | 492 (149-3195) | 605 (149-3195) | 447 (149-2224) | 0.267 |

**S2 Table. Hydration and Cardiovascular Profiles of the Participants on Extended Haemodialysis Prescriptions Stratified by their Overhydration Status.** bFGF= basic Fibroblast Growth Factor, BMI= Body Mass Index, BP= Blood Pressure, cm= centimetre, CRP= C-Reactive Protein, ECW= Extracellular Water, Flt-1 = Soluble fms-like tyrosine kinase-1, FTI = Fat Tissue Index, g= gram, HD= Haemodialysis, hr=hour, ICAM-1= Intercellular Adhesion Molecule-1, IL= Inteleukin, Kg= Killogram, L= Litre, LTI= Lean Tissue Index, m= meter, MAP= Mean Arterial Pressure, Median P50= red blood cell width (in micrometer), ml= millilitres, mmHg= millimetres of mercury, MMP= matrix metalloproteinase, ng= nanograms, OH = Overhydration Index, PBR= Perfused Boundary Region (in micrometers), pg=picogram, PIGF= Placenta Growth Factor, PWV = Pulse Wave Velocity, s= second, SAA= Soluble Amyloid A, Sig= Statistical Significance (p-value), TBW= Total Body Water, TNF= Tumour Necrosis Factor, VCAM-1= Vascular Cell Adhesion Molecule-1, VEGF= Vascular Endothelial Growth Factor, μg= microgram. ***** Highlights Result with statistical significance at the level of p<0.05.
