# Supplementary material for: Extracellular overhydration linked with endothelial dysfunction in the context of inflammation in haemodialysis dependent chronic kidney disease
Source: PLoS One. 2017 Aug 22;12(8):e0183281. doi: 10.1371/journal.pone.0183281 (PMC5568741; doi:10.1371/journal.pone.0183281)
Supplement: S3 Table — (DOCX) [file pone.0183281.s003.docx]

| Conventional HD Cohort  Characteristics | | Entire Cohort n=36 | No overhydration (OH/ECW < 7%) n=13 | Overhydration (OH/ECW > 7%) n=23 | Sig |
| --- | --- | --- | --- | --- | --- |
| Age (year) | | 62.1 (SD 13.8) | 59.6 (SD 18.6) | 63.4 (SD 10.4) | 0.503 |
| Sex: Male | | 28 (77.8%) | 9 (69.2%) | 19 (82.6%) | 0.345 |
| Ethnicity | **White** | 28 (77.8%) | 10 (76.9%) | 18 (78.3%) | 0.978 |
|  | **Black** | 5 (13.9%) | 2 (15.4 %) | 3 (13.0%) |  |
|  | **Asian** | 3 (8.3%) | 1 (7.7%) | 2 (8.7%) |  |
| Dialysis Vintage (months) | | 24.5 (6-106) | 22 (6-72) | 25 (6-106) | 0.262 |
| Residual Urine Output | | 20 (55.6%) | 8 (61.5%) | 12 (52.2%) | 0.587 |
| Previous Transplant | | 8 (22.2%) | 4 (30.8%) | 4 (17.4%) | 0.354 |
| Diabetes Mellitus | | 10 (27.5%) | 3 (23.1%) | 7 (30.4%) | 0.619 |
| CVD | | 14 (38.9%) | 6 (46.2%) | 8 (34.8%) | 0.501 |
| Smoking | | 8 (22.2%) | 2 (15.4%) | 6 (26.1%) | 0.458 |
| Davies Comorbidity Score | | 1 (0-4) | 1 (0-3) | 1 (0-4) | 0.219 |
| Number of BP medication | | 1.5 (0-5) | 1 (0-5) | 2 (0-5) | 0.426 |
| HD Parameters | **Hrs per wk** | 12 (10.5-12.0) | 12 (12-12) | 12 (10.5-12) | 0.180 |
|  | **HD frequency per wk** | 3 (3-4) | 3 (3-3) | 3 (3-4) | 0.281 |
|  | **HD session length** | 4 (3-4) | 4 (4-4) | 4 (3-4) | 0.116 |
|  | **Standard Kt/v (n=67)** | 2.16 (SD 0.20) | 2.19 (SD 0.16) | 2.13 (SD 0.22) | 0.429 |

**S3 Table. Conventional Haemodialysis Cohort Demographic and Dialysis Profiles**. BP= Blood Pressure, CVD= Cardiovascular Disease, ECW= Extracellular Water, HD= Haemodialysis, hr= hour, Kg= Kilogram, OH= Overhdration Index, SD= Standard Deviation, Sig= Statistical Significance (p-value). ***** Highlights Result with statistical significance at the level of p<0.05.
