# Supplementary material for: Extracellular overhydration linked with endothelial dysfunction in the context of inflammation in haemodialysis dependent chronic kidney disease
Source: PLoS One. 2017 Aug 22;12(8):e0183281. doi: 10.1371/journal.pone.0183281 (PMC5568741; doi:10.1371/journal.pone.0183281)
Supplement: S4 Table — (DOCX) [file pone.0183281.s004.docx]

| Fluid and  Cardiovascular Parameters | | Entire Conventional HD Cohort n=36 | No overhydration  (OH/ECW < 7%)  n=13 | Overhydration  (OH/ECW > 7%)  n=23 | Sig |
| --- | --- | --- | --- | --- | --- |
| Body Composition | **OH (L)** | **1.5 (-1.8-6.7)** | **0.0 (-1.8-1.2)** | **2.1 (0.9-6.7)** | **<0.001*** |
|  | **OH/ECW (%)** | **8.30 (-12.8-27.8)** | **0.0 (-12.8-6.7)** | **10.4 (7.0-27.8)** | **<0.001*** |
|  | **TBW (L)** | 37.55 (SD 6.10) | 36.3 (SD 6.9) | 38.3 (SD 5.6) | 0.342 |
|  | **ECW/TBW** | **0.49 (SD 0.03)** | **0.47 (SD 0.03)** | **0.50 (SD 0.02)** | **<0.001*** |
|  | **Weight (Kg)** | 80.0 (SD 14.7) | 79.4 (SD 17.7) | 80.4 (SD 13.2) | 0.856 |
|  | **BMI (Kg/m2)** | 27.23 (SD 4.65) | 27.0 (SD 5.8) | 27.4 (SD 4.0) | 0.815 |
|  | **LTI (Kg/m2)** | 12.9 (SD 2.6) | 12.9 (SD 3.2) | 12.8 (SD 2.3) | 0.583 |
|  | **FTI (Kg/m2)** | 13.6 (SD 5.0) | 13.9 (SD 5.8) | 13.4 (SD 4.6) | 0.767 |
| Visit BP (mmHg) | **Systolic** | 142.1 (SD 28.4) | 132.7 (SD 30.9) | 147.5 (SD 26.0) | 0.135 |
|  | **Diastolic** | 75.6 (SD 14.5) | 73.3 (SD 16.8) | 77.4 (SD 13.1) | 0.417 |
|  | **MAP** | 120.1 (SD 22.1) | 112.9 (SD 24.5) | 124.1 (SD 20.0) | 0.145 |
| PWV (m/s) (n=10:22) | | 10.03 (SD 2.89) | 9.7 (SD 2.04) | 10.2 (SD 3.22) | 0.641 |
| 24hr BP (mmHg) (n=12:21) | **Systolic BP** | 130.3 (SD 21.5) | 126.9 (SD 25.9) | 132.2 (SD 18.9) | 0.502 |
|  | **Diastolic BP** | 78.9 (SD 15.1) | 80.6 (SD 21.9) | 78.0 (SD 10.0) | 0.644 |
|  | **MAP** | 96.1 (SD 15.6) | 94.6 (SD 18.4) | 100.3 (SD 15.9) | 0.993 |
| Capillaroscopy | **PBR 5-25** | 2.00 (SD 0.26) | 1.95 (SD 0.31) | 2.02 (SD 0.23) | 0.430 |
|  | **PBR 5-9** | 1.10 (SD 0.10) | 1.02 (SD 0.14) | 1.00 (SD 0.08) | 0.620 |
|  | **PBR 10-19** | 2.14 (SD 0.27) | 2.12 (SD 0.33) | 2.15 (SD 0.25) | 0.742 |
|  | **PBR 20-25** | 2.58 (SD 0.44) | 2.40 (SD 0.38) | 2.68 (SD 0.44) | 0.065 |
|  | **Median P-50** | 11.06 (SD 2.21) | 11.45 (SD 2.69) | 10.83 (SD 1.91) | 0.434 |
| Vascular Biology Panel | **ICAM-1 (ng/ml)** | 426 (259-1345) | 459.0 (259-543) | 418.0 (269-1345) | 0.754 |
|  | **VCAM-1 (ng/ml)** | 1024.7 (SD 324.7) | 910.4 (SD 192.6) | 1089.4 (SD 368.0) | 0.113 |
|  | **E-selectin (ng/ml)** | 12.1 (SD 6.0) | 14.2 (SD 6.48) | 10.9 (SD 5.43) | 0.115 |
|  | **P-selectin (ng/ml)** | 45.2 (21.7-101.6) | 44.0 (21.7-88.4) | 46.3 (28.7-101.6) | 0.742 |
|  | **ICAM-3 (ng/ml)** | 1.1 (0.2-4.9) | 0.9 (0.2-4.9) | 1.1 (0.5-1.9) | 0.337 |
|  | **TM (ng/ml)** | 12.4 (8.3-20.1) | 11.6 (SD 2.31) | 13.2 (SD 3.56) | 0.161 |
|  | **MMP-1 (ng/ml)** | 32 (2-140) | 23 (10-105) | 33 (2-140) | 0.818 |
|  | **MMP-3 (ng/ml)** | 48 (13-255) | 45 (14-255) | 53 (13-112) | 0.307 |
|  | **MMP-9 (ng/ml)** | 111.5 (26-389) | 121.0 (100-267) | 78.0 (26 -389) | 0.068 |
| Pro-inflammatory Panel | **CRP (μg/ml)** | 4.3 (0.5-140.3) | 2.1 (0.6-68.1) | 5.4 (0.5-140.3) | 0.161 |
|  | **SAA (μg/ml** | 8.3 (0.7-165.4) | 9.9 (3.1-165.4) | 7.6 (0.7-143.0) | 0.469 |
|  | **IL6 (pg/ml)** | 1.9 (0.6-11.8) | 1.4 (0.8-7.4) | 2.3 (0.6-11.8) | 0.222 |
|  | **IL8 (pg/ml)** | 15.6 (4.5-185.7) | 15.5 (4.5-19.4) | 16.4 (7.8-185.7) | 0.374 |
|  | **TNF-α (pg/ml)** | 5.8 (4.2-12.0) | 5.8 (4.2-7.9) | 5.9 (4.2-12.0) | 0.531 |
| Growth Factors | **bFGF (pg/ml)** | 3.5 (0-28) | 4.0 (0-28) | 3 (0-13) | 0.767 |
|  | **PIGF (pg/ml)** | 24.5 (17-60) | 25.0 (17-45) | 24.0 (20-60) | 0.390 |
|  | **Flt-1 (pg/ml)** | 251.5 (147-1434) | 250.0 (155-396) | 252.0 (147-1434) | 0.754 |
|  | **VEGF (pg/ml)** | 605.5 (239-1732) | 640.0 (298-1361) | 561.0 (239-1732) | 0.791 |
|  | **Leptin (pg/ml)** | 8615 (338-169213) | 70918 (432-169213) | 7457 (338-162842) | 0.143 |
|  | **Insulin (pg/ml)** | 401 (0-2317) | 408 (131-2317) | 387 (0-2102) | 0.921 |

**S4 Table. Hydration and Cardiovascular Profiles of the Participants on Conventional Haemodialysis Prescriptions Stratified by their Overhydration Status.** bFGF= basic Fibroblast Growth Factor, BMI= Body Mass Index, BP= Blood Pressure, cm= centimetre, CRP= C-Reactive Protein, ECW= Extracellular Water, Flt-1= soluble fms-like tyrosine kinase-1, FTI = Fat Tissue Index, g= gram, HD= Haemodialysis, hr=hour, ICAM-1= Intercellular Adhesion, Molecule-1, IL= Inteleukin, Kg= Killogram, L= Litre, LTI= Lean Tissue Index, m= meter, MAP= Mean Arterial Pressure, Median P50= red blood cell width (in micrometer), ml= millilitres, mmHg= millimetres of mercury, MMP=matrix metalloprotainase, ng= nanograms, OH = Overhydration Index, PBR= Perfused Boundary Region (in micrometers), pg=picogram, PIGF= Placenta Growth Factor, PWV = Pulse Wave Velocity, s= second, SAA= Soluble Amyloid A, Sig= Statistical Significance (p-value), TBW= Total Body Water, TM= Thrombomodulin, TNF= Tumour Necrosis Factor, VCAM-1= Vascular Cell Adhesion Molecule-1, VEGF= Vascular Endothelial Growth Factor, μg= microgram. ***** Highlights Result with statistical significance at the level of p<0.05.
