# Supplementary material for: Extracellular overhydration linked with endothelial dysfunction in the context of inflammation in haemodialysis dependent chronic kidney disease
Source: PLoS One. 2017 Aug 22;12(8):e0183281. doi: 10.1371/journal.pone.0183281 (PMC5568741; doi:10.1371/journal.pone.0183281)
Supplement: S5 Table — (DOCX) [file pone.0183281.s005.docx]

| Potential Predictors of Overhydration | | Univariate Analysis | |
| --- | --- | --- | --- |
|  |  | OR (95% CI) | Sig |
| Age | | 1.04 (1.00-1.07) | 0.055 |
| Dialysis Vintage | | 1.00 (1.00-1.01) | 0.253 |
| Residual Urine Output | | 1.53 (0.55-4.26) | 0.418 |
| Diabetes Mellitus | | **20.00 (1.42- 282.45)** | **0.027*** |
| CVD | | 2.00 (0.67-6.01) | 0.217 |
| Smoking | | 1.08 (0.28-4.23) | 0.908 |
| Davies Comorbidity Score | | **1 .62 (1.06-2.48)** | **0.026*** |
| Number of BP medication | | 1 .41 (0.93-2.13) | 0.108 |
| HD Parameters | **Hrs per wk** | 0.99 (0.92-1.07) | 0.823 |
|  | **HD frequency per wk** | 0.84 (0.35-1.98) | 0.694 |
|  | **HD session length** | 1.03 (0.77-1.39) | 0.837 |
|  | **Standard Kt/v (n=67)** | 0.57 (0.17-1.94) | 0.367 |
| Body Composition | **LTI** | 0.96 (0.80-1.16) | 0.692 |
|  | **FTI** | 0.95(0.87- 1.04) | 0.294 |
| Visit BP | **Systolic** | **1.02 (1.00-1.04)** | **0.030*** |
|  | **Diastolic** | 1.01 (0.98-1.05) | 0.498 |
|  | **MAP** | **1.02 (1.00-1.05)** | **0.047*** |
| PWV (n=68) | | 1.11 (0.93-1.33) | 0.250 |
| 24hr BP (n=64) | **Systolic BP** | 1.02 (1.00-1.05) | 0.059 |
|  | **Diastolic BP** | 1.01 (0.98-1.05) | 0.509 |
|  | **MAP** | 1.02 (0.99-1.05) | 0.197 |
| Capillaroscopy | **PBR 5-25** | 1.03 (0.17-6.29) | 0.972 |
|  | **PBR 5-9** | 0.23 (0.002-24.91) | 0.540 |
|  | **PBR 10-19** | 0.46 (0.07-2.98) | 0.458 |
|  | **PBR 20-25** | 1.75 (0.67-4.60) | 0.255 |
|  | **Median P-50** | 0.86 (0.67-1.12) | 0.271 |
| Vascular Biology Panel | **ICAM-1** | 1.00 (1.00-1.01) | 0.239 |
|  | **VCAM-1** | **1.00 (1.00-1.01)** | **0.003*** |
|  | **E-selectin** | 0.93 (0.85 -1.02) | 0.107 |
|  | **P-selectin** | 1.00 (0.97-1.03) | 0.997 |
|  | **ICAM-3** | 0.93 (0.44-1.99) | 0.859 |
|  | **Thrombomodulin** | **1.19 (1.00-1.41)** | **0.047*** |
|  | **MMP-1** | 1.00 (0.89-1.02) | 0.974 |
|  | **MMP-3** | 1.00 (0.99-1.01) | 0.794 |
|  | **MMP-9** | 1.00 (0.99-1.01) | 0.892 |
| Pro-inflammatory Panel | **CRP** | 1.02 (0.99-1.05) | 0.134 |
|  | **SAA** | 1.01 (1.00-1.02) | 0.278 |
|  | **IL6** | **1.42 (1.06-1.92)** | **0.021*** |
|  | **IL8** | 1.07 (1.00-1.13) | 0.050 |
|  | **TNF-α** | 1.08 (0.88-1.33) | 0.480 |
| Growth Factor | **bFGF** | 0.99 (0.91-1.07) | 0.743 |
|  | **PIGF** | 1.02 (0.97-1.08) | 0.476 |
|  | **Flt-1** | 1.00 (1.00-1.00) | 0.703 |
|  | **VEGF** | 1.00 (1.00-1.00) | 0.419 |
|  | **Leptin** | **0.99 (0.98-0.998)** | **0.016*** |
|  | **Insulin** | 1.00 (1.00-1.00) | 0.267 |

**S5 Table. Assessment of Potential Predictors of Ovehydration.** bFGF= basic Fibroblast Growth Factor, BP= Blood Presssure, CI= Confidence Interval, CRP= C-Reactive Protein, CVD = Cardiovascular Disease, EF= Ejection Fraction, Flt-1= Soluble fms-like tyrosine kinase-1, HD= haemodialyis, hrs= hours, ICAM-1= Intercellular Adhesion Molecule-1, IL= Interleukin, LV= Left Ventricular, LVMI= Left Ventricular Mass Index, MAP= Mean Arterial Pressure, Median P50= Red Blood column width, MMP= Matrix Metalloproteinase, OR= Odd Ratio, PBR= Perfused Boundary Region of the endothelial glycocalyx, PIGF= Placenat Growth Factor, PWV= Pulse wave velocity, SAA= Soluble Amyloid A, Sig= Statistical Significance, TNF= Tumour Necrosis Factor, VCAM-1= Vascular Call Adhesion Molecule-1, VEGF= Vascular Endothelial Growth Factor, wk= week.
